# Summary of the DREAM8 Parameter Estimation Challenge: Toward Parameter Identification for Whole-Cell Models

**DOI:** 10.1371/journal.pcbi.1004096

**Published:** 2015-05-28

**Authors:** Jonathan R. Karr, Alex H. Williams, Jeremy D. Zucker, Andreas Raue, Bernhard Steiert, Jens Timmer, Clemens Kreutz, DREAM8 Parameter Estimation Challenge Consortium, Simon Wilkinson, Brandon A. Allgood, Brian M. Bot, Bruce R. Hoff, Michael R. Kellen, Markus W. Covert, Gustavo A. Stolovitzky, Pablo Meyer

**Affiliations:** 1 Graduate Program in Biophysics, Stanford University, Stanford, California, United States of America; 2 Department of Biology, Brandeis University, Waltham, Massachusetts, United States of America; 3 Volen Center for Complex Systems, Brandeis University, Waltham, Massachusetts, United States of America; 4 Broad Institute of MIT and Harvard, Cambridge, Massachusetts, United States of America; 5 Institute for Physics, University of Freiburg, Freiburg, Germany; 6 Freiburg Center for Systems Biology (ZBSA), University of Freiburg, Freiburg, Germany; 7 Freiburg Institute for Advanced Studies (FRIAS), University of Freiburg, Freiburg, Germany; 8 BIOSS Centre for Biological Signalling Studies, University of Freiburg, Freiburg, Germany; 9 Numerate Inc., San Bruno, California, United States of America; 10 Sage Bionetworks, Seattle, Washington, United States of America; 11 Department of Bioengineering, Stanford University, Stanford, California, United States of America; 12 IBM Thomas J. Watson Research Center, Yorktown Heights, New York, United States of America; University of Washington, UNITED STATES

## Abstract

Whole-cell models that explicitly represent all cellular components at the molecular level have the potential to predict phenotype from genotype. However, even for simple bacteria, whole-cell models will contain thousands of parameters, many of which are poorly characterized or unknown. New algorithms are needed to estimate these parameters and enable researchers to build increasingly comprehensive models. We organized the Dialogue for Reverse Engineering Assessments and Methods (DREAM) 8 Whole-Cell Parameter Estimation Challenge to develop new parameter estimation algorithms for whole-cell models. We asked participants to identify a subset of parameters of a whole-cell model given the model’s structure and in silico “experimental” data. Here we describe the challenge, the best performing methods, and new insights into the identifiability of whole-cell models. We also describe several valuable lessons we learned toward improving future challenges. Going forward, we believe that collaborative efforts supported by inexpensive cloud computing have the potential to solve whole-cell model parameter estimation.

## Author Summary

Whole-cell models promise to enable rational bioengineering by predicting how cells behave. Even for simple bacteria, whole-cell models require thousands of parameters, many of which are poorly characterized or unknown. New approaches are needed to estimate these parameters. We organized the Dialogue for Reverse Engineering Assessments and Methods (DREAM) 8 Whole-Cell Parameter Estimation Challenge to develop new approaches for whole-cell model parameter identification. Here we describe the challenge, the best performing methods, new insights into the identifiability of whole-cell models, and several lessons we learned for improving future challenges. Going forward, we believe that collaborative efforts have the potential to produce powerful tools for identifying whole-cell models.

## Introduction

Mechanistic modeling is a powerful tool for understanding and engineering biological behavior at the molecular level. Davidson et al. have used Boolean modeling to understand *Drosophila* developmental patterning [1]; Orth et al. have used flux-balance analysis (FBA) to predict *Escherichia coli* metabolism at the genomic scale [2]; Barkai and Leibler have used ordinary differential equations (ODEs) to model *E*. *coli* chemotaxis [3]; Arkin et al. have used stochastic ODEs to understand the bacteriophage *λ* lysis/lysogeny switch [4]; and many others have used mechanistic models to study a wide range of cell physiology. Despite these successes, no one mathematical formalism is capable of explaining all biological behaviors. Consequently, a comprehensive predictive understanding of biology behavior has remained elusive.

Recently, Karr et al. developed an integrative modeling approach that enabled them to construct the first whole-cell model by combining submodels of 28 cellular processes [5]. This approach enabled them to model each process using the most appropriate mathematics. For example, they modeled metabolism using FBA [6] and cytokinesis using ODEs. Mathematically, the model is a stochastic, discrete–continuous hybrid, nonlinear, dynamical system. Furthermore, the model is computationally expensive.

The model accounts for the function of every annotated gene product of the gram-positive bacterium *Mycoplasma genitalium* and predicts the dynamics of every molecular species. The model has enabled researchers to gain insights into cell cycle regulation, as well as to predict kinetic parameters [7].

Predictive models begin with a list of molecular components [8]. This can be captured using unbiased high-throughput experiments including DNA sequencing and mass spectrometry. Molecular components are then connected through interactions into wiring diagrams. These interactions can be assembled from prior knowledge or inferred from high-throughput experiments such as microarrays or flow cytometry [9–12]. Next, wiring diagrams are translated into quantitative mathematical models. This introduces quantitative parameters such as transition probabilities, reaction turnover numbers, and binding affinities. Lastly, parameter values are curated from prior knowledge or estimated from experimental data.

Accurate parameter values are essential for reliable prediction [13]. Unfortunately, many parameters have not been characterized. Consequently, parameter estimation is critical for model construction.

In principle, parameters can be estimated using numerical optimization. Many techniques are available, including derivative-based initial value methods and stochastic multiple shooting methods [14]. However, few techniques are tractable for computationally expensive models. Numerical optimization must be combined with additional techniques such as surrogate modeling, model reduction, distributed optimization, or automatic differentiation.

Surrogate modeling and model reduction minimize the computational cost of optimization by replacing the original function with a cheaper, approximate function [15–18]. Surrogate modeling, which is also referred to as function approximation, metamodeling, response surface modeling, and model emulation, uses statistical models including artificial neural networks, splines, and support vector machines. Model reduction uses lower fidelity physical models. Surrogate modeling and model reduction have been used in several fields, including aerospace engineering [19], hydrology [20], and petroleum engineering [21].

Distributed optimization is also a promising approach for optimizing computationally expensive models. It uses multiple agents, each simultaneously employing the same algorithm on different regions, to quickly identify optima [22,23]. Typically, agents cooperate by exchanging information so that agents learn from each other’s experiences. Distributed optimization has also been used in several fields including aerospace and electrical engineering [24,25] and molecular dynamics [26].

Another potential approach for optimizing computationally expensive models is automatic differentiation, an efficient technique for analytically computing the derivative of a computational model by decomposing the model into elementary functions to which the chain rule can be applied [27]. Automatic differentiation can be used to make derivative-based optimization methods tractable in cases where finite difference calculations are prohibitively expensive. It has been used to identify parameters in chemical engineering [28], biomechanics [29], and physiology [30].

Estimating the parameters of whole-cell models is further complicated by limited experimental data, stochastic variation, and measurement error [14]. Taken together, parameter estimation is an important problem in systems biology, as researchers pursue increasingly comprehensive and accurate models.

We organized the Dialogue for Reverse Engineering Assessments and Methods (DREAM) 8 Whole-Cell Parameter Estimation Challenge to develop new parameter estimation methods for whole-cell models. Stolovitzky and Califano founded DREAM to foster collaborative efforts by computational and experimental biologists to reverse engineer cellular networks from high-throughput data [31]. DREAM challenges have repeatedly demonstrated the “wisdom of crowds” to produce high-quality methods [32–34]. This challenge focused on developing and assessing methods for estimating parameters of computationally expensive hybrid mathematical models. Previous challenges, in contrast, have focused on lower dimensional models, although some previous challenges have asked participants to estimate more parameters [35].

To mimic real-life whole-cell model parameter estimation, we challenged participants to identify a subset of parameters of a slow-growing mutant in silico strain of a recent whole-cell model of *M*. *genitalium*. We created the mutant strain by modifying its parameters to increase its predicted doubling time. We used the mutant strain to simulate several commonly available experimental datasets.

We encouraged participants to form teams and gave participants 15 weeks to identify the unknown parameters. We provided participants the model’s structure, its wild-type strain parameter values, and mutant strain in silico “experimental” data. We also allowed participants to obtain a limited amount of perturbation data. This was designed to mimic the real-life scenario of limited experimental resources and encourage participants to identify the most informative data types and perturbations.

To foster collaboration among teams, we divided the competition into four subchallenges and required teams to share their methodology to compete in each subchallenge. To maximize participation, we provided participants the BitMill cloud computing service (http://bitmill.numerate.com) to evaluate the model. Participants used BitMill to calculate model predictions and errors. Unintentionally, BitMill also provided information about the distance between the submitted and true parameter values, which is never available in real-world parameter estimation. Unfortunately, we were not alerted to this mistake until the end of the challenge, at which point it was too late to change the challenge.

Ten teams participated in the challenge. Six teams pursued the true parameter estimation problem using only the training data and the prediction errors computed by BitMill. Four teams also used the parameter errors returned by BitMill, instead focusing on an artificial parameter estimation problem. The teams used a variety of parameter estimation techniques. All of the teams, including those that focused on the artificial parameter estimation problem, generated valuable ideas about how to best identify whole-cell models and about the tractability of the true parameter estimation problem.

Here we describe the challenge setup and the top performing methods. We examine the submissions to identify the most identifiable parameters and reproducible predictions. We conclude by discussing the remaining obstacles to identifying whole-cell models and by describing how to improve future challenges.

## Methods

### *M*. *genitalium* Whole-Cell Model

We asked participants to identify a modified model of the gram-positive bacterium *M*. *genitalium* [5]. The model is composed of submodels of 28 cellular processes, each of which was modeled independently at short time scales using different mathematical representations. For example, the metabolism submodel was modeled using FBA, whereas the transcription submodel was modeled using stochastic methods. The submodels were integrated through 16 cell state variables that represented the instantaneous configuration of the cell and its external environment, including metabolite, RNA, and protein copy numbers, reaction fluxes, nascent RNA and protein sequences, and DNA-binding protein locations. Mathematically, the model is a stochastic, discrete–continuous hybrid, nonlinear, dynamical system.

Each model simulation predicts the dynamics of each molecular species over the life cycle of one in silico cell. Each simulation requires approximately one core day.

### Wild-Type Model Parameters

The whole-cell model contains 1,462 quantitative parameters including average metabolite concentrations, RNA polymerase promoter binding affinities, RNA half-lives, and reaction kinetics (S1 Table). The wild-type values of these parameters were initially set according to published experimental measurements.

However, the model’s predictions based on these initial values were inconsistent with the measured doubling time. Consequently, Karr et al. modified the model’s parameters to match the physiological data. Numerical optimization methods that require large numbers of model evaluations were prohibitively expensive. Instead, Karr et al. optimized the model’s parameters using a reduced model.

First, Karr et al. constructed a reduced physical model that approximates the temporal and population average of the full model. The reduced model has the same parameters as the full model, but is computationally cheaper. Second, they minimized the reduced model’s prediction error by numerically optimizing its parameters. Next, they calculated the full model’s prediction error with the optimized parameter values. Lastly, they manually tuned the full model’s parameters to reduce its prediction error. Their model reduction approach is described in Data S1 of Karr et al., 2012 [5].

### Mutant In Silico Strain

We challenged participants to identify an in silico mutant strain with a significantly altered phenotype from that of the wild-type strain. Because the original model was primarily used to investigate the molecular determinants of the growth rate, we decided to ask participants to identify a slow-growing mutant strain. To limit the difficulty of the challenge, we decided to modify only 15 parameters. The precise number of modified parameters was chosen arbitrarily. Furthermore, we only modified three types of parameters: the RNA polymerase promoter binding probabilities and RNA half-lives, which control RNA expression and in turn metabolic enzyme expression, and the metabolic reaction turnover numbers. We focused on these three types of parameters because these parameters uniquely map onto changes in specific observables and are therefore structurally identifiable, and because these parameters have the most direct influence on the metabolic submodel, and in turn the predicted growth rate.

We constructed the mutant in silico strain by modifying a subset of the model’s parameter values. First, we calculated the sensitivity of the predicted doubling time to the RNA polymerase binding probabilities, RNA half-lives, and reaction turnover numbers. Second, we used the sensitivities to estimate the parameter value changes required to increase the predicted doubling time by 1.9%. We chose 1.9% so that iteratively modifying the 15 parameters would together increase the predicted doubling time by 33%. Third, we randomly selected a single parameter to modify, weighted by its estimated fold value changes from the previous step. Next, we modified the value of the selected parameter. We iteratively repeated this to achieve a mutant strain with a 33% increased doubling time.

The mutant strain construction procedure selected three polymerase promoter binding probabilities, three RNA half-lives, and nine metabolic reaction turnover numbers. The procedure increased the values of two of these parameters 3%–95% and decreased the values of the remaining 13 12%–91%.

To further limit the difficulty of the challenge, we told participants the identities of the 15 modified parameters plus the identities of 15 additional unmodified parameters of the same three types (S2–S4 Tables). This was designed to increase the tractability of the challenge by reducing the dimensionality of the search space, as well as to determine if the participants were able to distinguish between modified and unmodified parameters. The precise number of unknown parameters was chosen arbitrarily.

### In Silico “Experimental” Data

We constructed eight sets of in silico “experimental” data for parameter estimation. These mimicked the experimental data available for real-world parameter estimation. They included one single-cell data set: growth, mass, and volume time courses and replication initiation, replication, and cytokinesis times. They also included seven temporal and population average data sets: metabolite concentrations, DNA-seq, RNA-seq, ChIP-seq, RNA expression arrays, protein expression array, and metabolic reaction fluxes.

We simulated the eight in silico data sets for the mutant strain, as well as for 2-fold up and down perturbations to each of the 30 unknown parameters. Each mutant strain data set was simulated using a population of 32 in silico cells; each perturbation data set was simulated using eight cells. In total, we simulated eight mutant strain data sets and 480 perturbation data sets.

The eight data sets were chosen such that each of the unknown parameters were expected to be practically identifiable. The ChIP-seq data contains information about the unknown RNA synthesis rates, together the ChIP-seq and RNA half-life data contain information about the RNA synthesis rates, and the reaction flux data contains information about the metabolic kinetic rates. It is important to note that the unknown parameters would have been substantially more difficult to identify with the scalar prediction error alone. The in silico data sets contain valuable information for parameter identification.

We provided participants all eight mutant strain data sets. In addition, to mimic the real-life scenario of limited experimental resources, we allowed participants to obtain up to 50 perturbation data sets.

### BitMill Cloud-Computing Service

We provided participants the BitMill cloud computing service to simulate the in silico data sets and calculate prediction errors. To ensure equal access to BitMill, we limited participants to eight simultaneous simulations during the first ten weeks and 40 during the final five weeks.

### Teams

To mimic real-life collaborative research, we created an online forum to help participants find teammates. Teams were allowed to pool in silico perturbation data and BitMill resources.

### Subchallenges and Scoring

To foster collaboration among teams, we divided the competition into four subchallenges and required participants to share their methodology to compete in each subchallenge. This enabled teams to learn from the best performing methods throughout the challenge.

For the first subchallenge, we ranked submissions by their log ratio parameter error,

$$e_{\text{param}}=\frac{1}{N}\sum_{i=1}^{N}{\left({\text{log}}_{10}\frac{v_{i}^{\text{est}}}{v_{i}^{\text{true}}}\right)}^{2}\;,$$ (1)

where $v_{i}^{\text{true}}$ and $v_{i}^{\text{est}}$ are the true and estimated parameter values, and *N* = 30 is the number of unknown parameters. For the third subchallenge, we ranked submissions by their least squares prediction error,

$$e_{\text{predict}}=\frac{1}{M}\sum_{i=1}^{M}{\left(\frac{v_{i}^{\text{true}}-v_{i}^{\text{est}}}{\sigma_{i}^{\text{true}}}\right)}^{2}\;,$$ (2)

where $v_{i}^{\text{true}}$ and $v_{i}^{\text{est}}$ are the true and estimated values of simulated experimental measurement *i*, $\sigma_{i}^{\text{true}}$ is the true variance of measurement *i*, and *M* = 2,810,064 is the total number of simulated experimental measurements. We judged the creativity of the participants’ methodologies for the second subchallenge.

We scored the final challenge by combining the parameter and prediction errors used for the first and third subchallenges. First, we calculated the parameter and prediction *p*-values of each submission, *p*_param_ and *p*_predict_, using empirical parameter and prediction error distributions. We constructed these empirical distributions by calculating the errors of meta parameter and prediction vectors formed by randomly sampling the submitted parameter vectors and simulated prediction vectors [35]. Next, we computed an overall score, *s*, by combining the parameter and prediction *p*-values multiplicatively,

$$s=-\text{ln}\;p_{\text{param}}p_{\text{predict}}.$$ (3)

### Prizes

We motivated participants to compete in the final subchallenge by offering winners the opportunity to present their methodology at the annual Research in Computational Molecular Biology (RECOMB) Conference on Regulatory and Systems Genomics and in this manuscript. In addition, we offered small cash awards, scientific software, and other small prizes for the winners of the first three subchallenges.

### Challenge Organization

We organized the challenge using the Synapse workspace (https://www.synapse.org/#!Synapse:syn1876068). We used Synapse to distribute challenge materials, administer the perturbation data, collect submissions, and announce winners. We used GitHub (http://github.com/CovertLab/WholeCell/tree/parameter-estimation-DREAM-challenge-2013.) to distribute the model to participants. We used a Get Satisfaction forum (http://getsatisfaction.com), GoToWebinar (http://www.gotomeeting.com), and YouTube to communicate with participants through a webinar (https://www.youtube.com/watch?v=VQA9YwsAgQk).

## Results

### Participation

Ten teams comprising 45 researchers from 16 institutions and six countries participated in the challenge. The researchers represented a broad variety of disciplines, including biology, computer science, mathematics, physics, and statistics. The researchers also spanned a wide range of experience levels ranging from undergraduate students to senior faculty. In total, nine teams submitted 691 solutions, including 682 solutions from the five top performing teams. One team obtained all of the perturbation data and performed simulations on their own computers, but did did not submit a solution.

### Perturbation Data Usage

Three teams collected 586 perturbation experiments. One of the top four teams collected all 60 single-cell data sets, as well as 19 of 20 metabolic reaction flux and DNA-seq measurements of increased turnover numbers. A second team collected all 20 metabolic reaction flux measurements of perturbed turnover numbers. A third team collected all 480 data sets. However, this team did not submit any solutions. Surprisingly, seven teams did not collect any perturbation data, including four of the top five teams.

Overall, participants used the perturbation data minimally. Only two of nine teams that submitted solutions obtained perturbation data. Both of these teams focused on the metabolic turnover rate perturbations and metabolomic data, possibly because the mutant strain exhibited a metabolic, slow-growth phenotype. However, neither team discussed the perturbation data in their write-ups. Together, this suggests that teams did not use experimental design strategies to focus on the most likely informative data, or use the data to estimate parameters. This contrasts what has been observed in other DREAM challenges for smaller models [35]. Instead, the model’s stochasticity led most of the teams to focus on generating more precise training data by running and averaging large numbers of their own simulations.

### Cloud Computing Usage

Participants used the BitMill cloud computing service extensively. During the first 10 weeks when participants were limited to eight simultaneous simulations, participants requested 100 simulations per week. Participants submitted simulations 5-fold more frequently after the BitMill limit was increased 5-fold at the end of the tenth week. We believe that BitMill was critical to the success of the challenge.

### Parameter Estimation Performance

**Fig 1:**
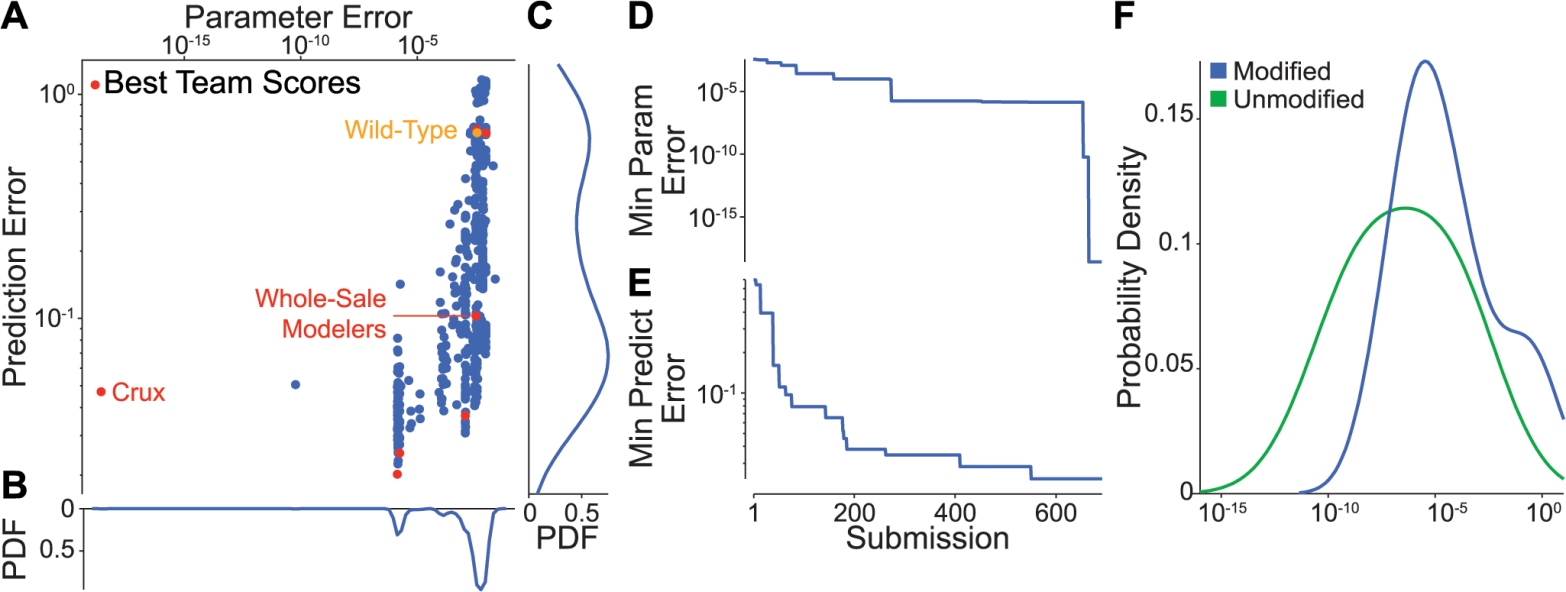
Overview of participant performance. Nine actively competing teams submitted 691 submissions, including 682 submissions from the five most active and top performing teams. **A**: Submission error distribution. Red indicates the top scoring submissions for each of the nine active teams. For comparison, orange indicates the parameter and prediction errors of the wild-type parameter values. Overall, the parameter and prediction errors are moderately correlated (log-log *R* ^2^ = 0.57). However, the errors are not correlated among the top scoring solutions of the five most active and best performing (log-log *R* ^2^ = 0.04). **B, C**: Marginal parameter and prediction error distributions. **D, E**: Progression of the minimum parameter and prediction errors across all teams during the challenge. Errors highlight steady participant improvement throughout the 15 week challenge. **F**: Error distribution of the 30 individual unknown parameters. Errors are averaged across the top 50 scoring solutions from all participants. Blue indicates the 15 modified parameters; green indicates the 15 unknown and unmodified parameters. The comparatively smaller errors for the unmodified parameters indicate that the participants correctly identified which parameters were modified. The overall small errors indicate that the participants ultimately identified the modified parameter values. However, not all parameters were equally well estimated.

Nine teams submitted 691 solutions, including 682 solutions from the five most active and top performing teams. We began analyzing the submissions by inspecting the distribution of parameter and prediction errors across all 691 solutions (Fig 1A). Interestingly, we found that although participants were able to reduce the parameter error by over 18 orders of magnitude from the wild-type parameter values, they were only able to reduce the prediction error 30-fold. As discussed below, several participants were able to perform substantially better on the parameter error metric than on the prediction error metric by using information to directly minimize the parameter error rather than indirectly minimizing the parameter error using the prediction error as a proxy.

We also found that the parameter and prediction errors are only moderately correlated (log–log *R* ^2^ = 0.57). This is primarily because the prediction error is sensitive to the model’s stochastic variation. Importantly, this suggests that the prediction error must be evaluated over a large number of model simulations to minimize its sensitivity to stochastic variation. Unfortunately, this magnifies the large computational cost of whole-cell model parameter estimation. The moderate correlation is also due in part to practical parameter unidentifiability given the limited training data, both in terms of phenotypic diversity and small numbers of samples, and therefore large stochastic variation. Interpreted biologically, this means that multiple sets of parameters can produce different molecular phenotypes but have similar systems-level phenotypes. Fortunately, this practical unidentifiability can typically be overcome for whole-cell models by using additional types of training data, which contain additional molecular information. For example, participants who only used the RNA-seq data, which provides information about the product of RNA synthesis rates and half-lives, would have found these parameters practically unidentifiable. However, participants who also used the ChIP-seq, which provides information about RNA synthesis rates, would have found these parameters identifiable. In the context of real-world whole-cell modeling research, an easy way to make parameters more identifiable is to collect additional molecular data which provides information about individual parameters. For example, an easy way to estimate RNA half-life parameters is to measure the decay rate of each individual RNA species. In contrast, additional systems level data typically does not significantly increase the practical identifiability of whole-cell models.

Next, we examined the participants performance over the duration of the challenge (Fig 1D and 1E). Despite the formidable difficulty of the challenge, we found that performance improved throughout the challenge. Notably, we observed that participants improved their parameter performance by over 13 orders of magnitude between submissions 654 and 666. Reviewing the participants’ write-ups, we learned that the dramatic improvement was due to a change in parameter estimation strategy by Team Crux (see below). Furthermore, the dramatic improvement occurred with little concomitant decrease in the prediction error, underscoring the weak correlation between the parameter and prediction errors.

Ultimately, primarily using the parameter error information, participants accurately identified the parameters. Table 1 lists each team’s methodology and performance.

**Table 1:** Team methods, parameter and prediction errors, and overall scores.

Team	Optimization method	Cost function	Reduction strategy	Estimation problem	Parameter error	Prediction error	Score
**Crux**	Derivative-based	MLE	*None*	Artificial	2.60×10^−19^	*0*.*052*	*38*.*1*
**New Dream**	*N/R*	*N/R*	*N/R*	Artificial	1.39×10^−6^	*0*.*023*	*11*.*6*
**ICM Poland**	Derivative-based	Log ratio (Eq 1)	*None*	Artificial	1.75×10^−6^	*0*.*028*	*9*.*35*
**Alucinatori**	Derivative-based	Log ratio (Eq 1)	*None*	Artificial	1.14×10^−3^	*0*.*041*	1.84×10^−4^
**Whole-Sale Modelers**	Differential evolution	Least squares (Eq 2)	Principal components	True	3.61×10^−3^	0.111	1.40×10^−4^
**CU**	*N/R*	*N/R*	Model reduction	True	3.56×10^−3^	*0*.*688*	6.71×10^−5^
**Team 9**	*N/R*	*N/R*	*N/R*	True	3.56×10^−3^	*0*.*711*	6.61×10^−5^
**Hurricane**	*N/R*	*N/R*	*N/R*	True	8.87×10^−3^	*0*.*689*	2.96×10^−5^
**DBI-Guesstimators**	*N/R*	*N/R*	*N/R*	True	8.87×10^−3^	*0*.*689*	2.96×10^−5^
**Uniandes**	*N/R*	*N/R*	*N/R*	True			

“Estimation problem” column indicates which teams used the parameter error data. Teams are listed by overall score in descending order. Team Uniandes did not submit a solution and therefore was not scored. Not reported (N/R) indicates teams that did not report their approach.

Next, we inspected the individual contributions of the unknown parameters to the parameter errors (Fig 1F). We found that the error distribution of the unknown, unmodified parameters is centered over two orders of magnitude left of that of the modified parameters, indicating that participants successfully differentiated the unmodified and modified parameters. More importantly, we found that the error distribution was very broad, suggesting that the parameters are unequally practically identifiable. As discussed below, this is likely because the predicted phenotypes are unequally sensitive to the parameters. Going forward, this suggests that broader phenotypic profiling is needed to identify whole-cell models.

**Fig 2:**
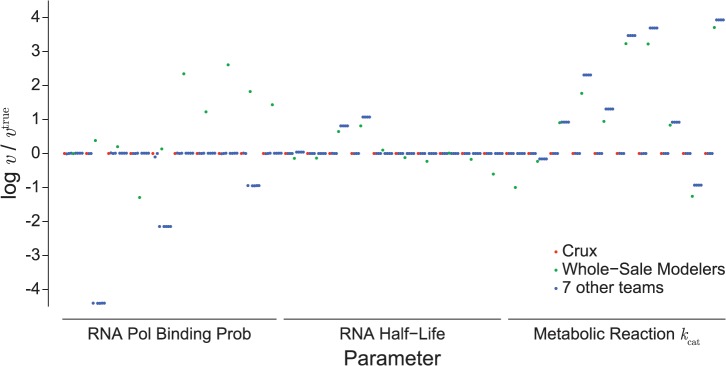
Estimation performance of individual parameters. log_2_ ratios of estimated and true mutant values of each unknown parameter. Red and green circles indicate Team Crux’s and Team Whole-Sale Modelers’ highest scoring solutions, respectively. Blue circles indicate the highest scoring solutions of the seven other teams.

To gain additional insight into the broad distribution of individual parameter errors, we plotted the ratio of each parameter’s true and predicted values for each team’s top scoring solution (Fig 2). This showed that Team Crux identified every parameter. Moreover, the analysis showed that teams had the most difficulty estimating the metabolic reaction turnover rates. Teams likely had the most difficulty estimating these parameters because they were changed significantly relative to the wild-type values and because they affect the in silico data nonlinearly. This suggests that additional types of experimental data that respond linearly to the turnover rates may improve turnover rate estimation.

### Phenotype Prediction Performance

**Fig 3:**
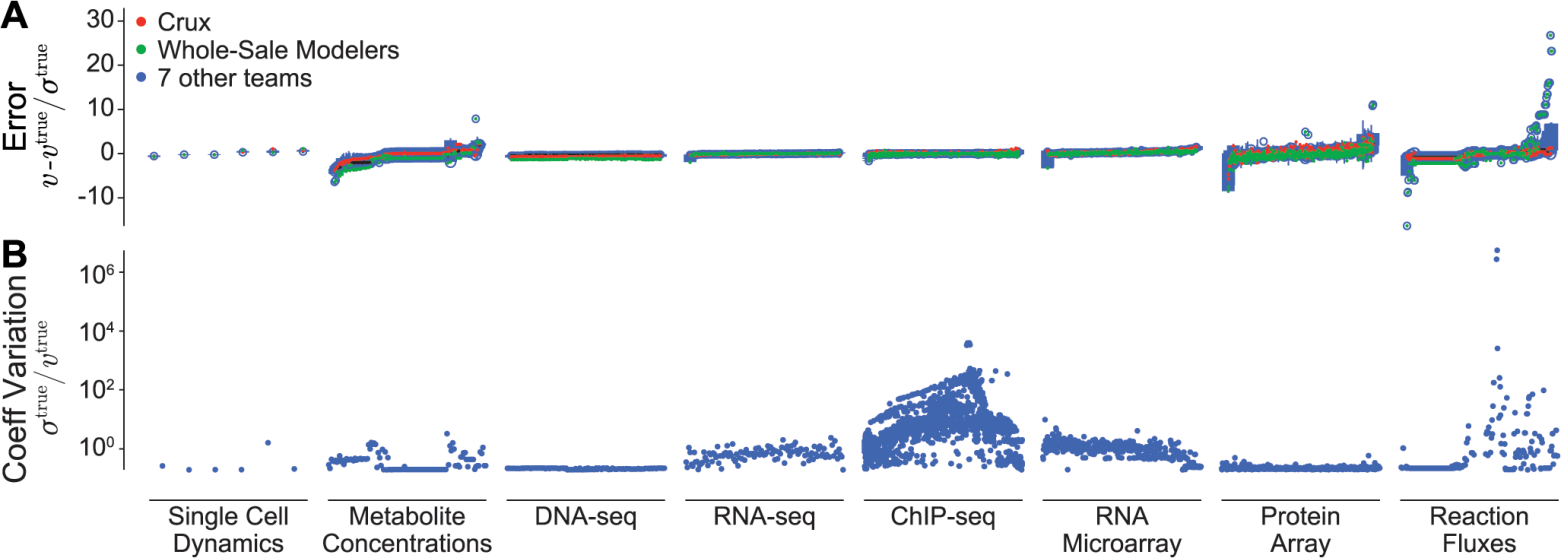
Individual phenotype prediction performance. **A**: Prediction performance of the top scoring solutions for the eight in silico data sets. Red and green circles indicate Team Crux’s and Team Whole-Sale Modelers’ highest scoring solutions, respectively. Blue circles indicate the highest scoring solutions of the seven other teams. **B**: Coefficients of variation of the eight in silico data sets across the life cycles of 32 mutant strain cells. This figure indicates that the reaction flux and ChIP-seq measurements are the most variable, meaning that individual in silico cells with identical parameter values stochastically exhibit significantly different metabolic reaction fluxes and protein-DNA binding patterns. This suggests that these measurements might be the most difficult to reproduce and the least informative for parameter identification.

Next, we analyzed the participants’ prediction performance of the individual in silico phenotypes (Fig 3A). We found that even the top scoring solutions produced some phenotypes that differed by more than 25 standard deviations from that of the mutant strain. In particular, we found that participants had difficulty reproducing several of the mutant strain reaction fluxes, protein expression values, and metabolite concentrations. We believe this is because these phenotypes are not only highly sensitive to the modified parameters but also highly variable and thus poorly sampled by the small number of simulations. Surprisingly, we also found that participants were able to reproduce some of the most variable in silico data including the ChIP-seq data (Fig 3B). This is because although the ChIP-seq data is highly variable across individual cells, it is relatively insensitive to the modified parameters and thus can be predicted relatively easily. In contrast, some of the least variable phenotypes, including the protein expression data, were difficult to reproduce because they are highly sensitive to modified probabilities and half-lives. Overall, the fact that participants had trouble reproducing the mutant phenotype, even with the help of parameter error metric, implies that whole-cell model parameter estimation requires large numbers of simulations to accurately compare model predictions and experimental training data. In turn, this means that whole-cell parameter estimation methods must be highly computationally efficient.

### Parameter Estimation Strategies

Broadly, participants used two families of strategies: (1) participants tried to solve the real-world problem of estimating the unknown parameter values using only the mutant and perturbation experimental data and the prediction error metric, and (2) participants tried to solve the artificial problem of identifying the parameters primarily using the parameter error. Initially, all teams pursued the first class of strategies. Together, they employed a variety of techniques including differential evolution and derivative-based approaches, as well as manual tuning guided by mathematical and biological intuition (Table 1). Table 2 summarizes the advantages and disadvantages of the methods used by the participants. Team Whole-Sale Modelers submitted the top scoring solution from this first class of strategies using an innovative technique combining differential evolution with random forests (Box 1).

**Table 2:** Comparison of employed parameter estimation methods.

Method	Advantages	Disadvantages
**Derivative-based**	Very efficient for convex functions	Sensitive to starting point; trapped by local maxima; sensitive to noise
**Differential evolution/random forests**	Insensitive to starting point; able to identify global maxima in complex landscapes; reports multiple high scoring solutions; less sensitive to noise; easily parallelizable; less computationally expensive	Inefficient for simple, convex functions
**Model reduction**	Efficient for computationally expensive models; reduced model has clear physical interpretation	Requires high fidelity reduced model; no general procedure for model reduction
**Statistical surrogate**	Efficient for computationally expensive models; surrogate can be constructed automatically	Many model evaluations required to construct surrogate; surrogate has no physical interpretation

**Box 1. Best Performing Prediction Error Method: Hybrid Differential Evolution-Random Forests Parameter Inference (Team Whole-Sale Modelers)**

Team Whole-Sale Modelers identified parameter sets that minimized the prediction error (Eq 2) by trading off exploration of the parameter space with exploitation of the phenotype measurements using a modified version of differential evolution (DE). DE is a population-based metaheuristic optimization method that minimizes a cost function by iteratively refining a population of solutions, (*x*_1_, *x*_2_, …), by only reproducing the fittest individuals [36]. DE is commonly used in many scientific fields including systems biology [37–39]. Team Whole-Sale Modelers used DE for several reasons. First, DE can identify global optima in nonlinear, multimodal landscapes such as that of the whole-cell model prediction error. Second, DE is robust to noise. Third, DE avoids prohibitively expensive computations such as the gradient of the prediction error.

**Fig 4:**
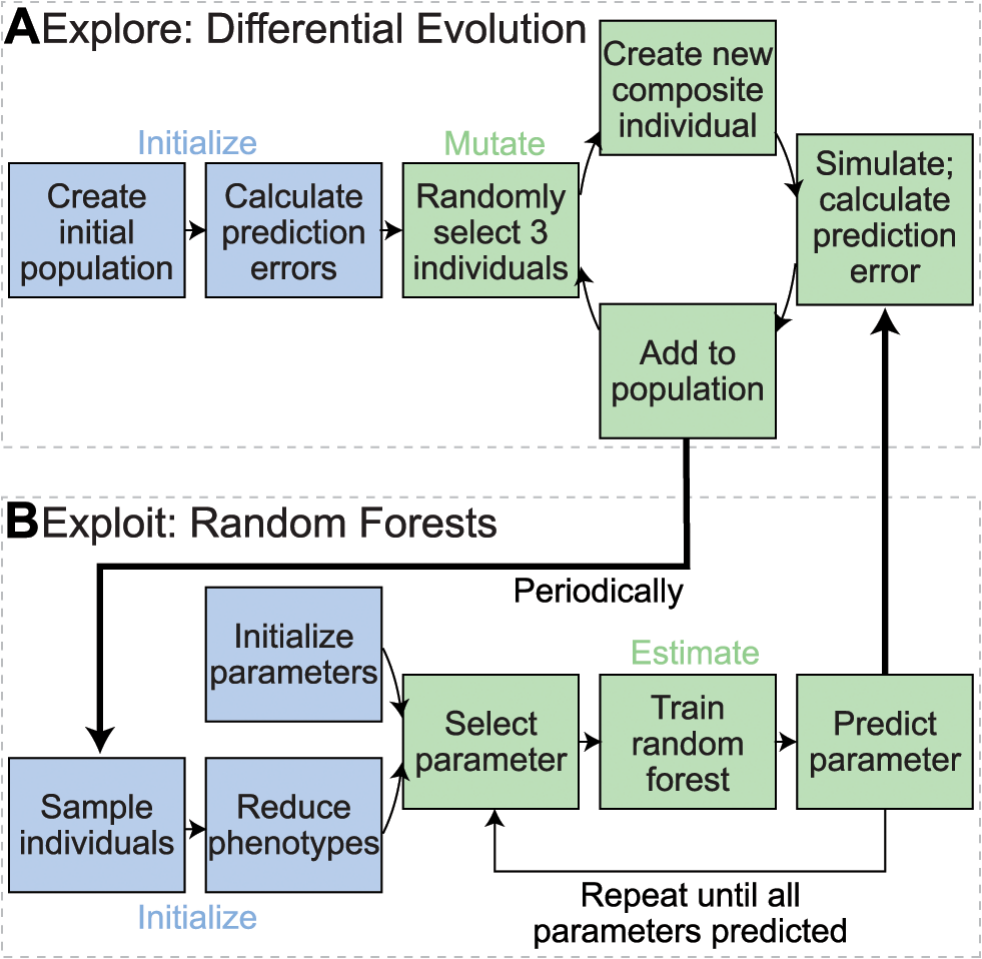
Team Whole-Sale Modelers iterative random forest parameter estimation method. Team Whole-Sale Modelers estimated the unknown parameters using a combination of differential evolution to explore the parameter space and random forests to exploit the available phenotype measurements. **A.** In the exploration phase, Team Whole-Sale Modelers generated an initial population of parameter estimates, and computed their prediction error relative to the mutant phenotype. Three individuals were then randomly selected without replacement from the population, and recombined using differential evolution (DE) to create new individuals. **B.** Periodically, Team Whole-Sale Modelers used random forests to add new individuals to the population. This was designed to exploit deeper structure in the phenotype measurements of the population. First, Team Whole-Sale Modelers used principal component analysis to create a training set that accounted for the majority of the variance in the phenotype measurements. Then, they used the first principal components to iteratively train random forest estimators for each individual parameter. After all of the parameters were estimated, they added the estimated parameter set to the DE population.

Team Whole-Sale Modelers employed DE to explore the parameter space and minimize the prediction error (Fig 4). Individuals represented multiplicative changes in the values of the unknown parameters. The population was initialized to 200 individuals. The mutation step was implemented by (1) randomly selecting three individuals *x*_*p*_, *x*_*q*_, and *x*_*r*_ from the population without replacement, (2) generating new individuals, *x*_new_, using the rule *x*_new_ = *x*_*p*_ + *F*(*x*_*q*_ – *x*_*r*_), where *e*_predict_ (*x*_*q*_) < *e*_predict_ (*x*_*r*_) and *F* is a random scalar between 0.2 and 1, and (3) bounding each dimension of the new individuals to between 0.066 and 1.906 according to the stated parameter change limits. New individuals were added to the population if *e*_predict_ (*x*_new_) was less than the median prediction error across all individuals. DE was run without the crossover step.

Team Whole-Sale Modelers periodically added individuals to the DE population by exploiting deeper structure in the phenotype measurements. The choice of exploitation algorithms was driven by several design constraints. First, due to the large computational cost, the number of sample points in the training set was quite small. Second, the number of phenotype measurements associated with each sample point was quite large. To reduce the dimensionality of the feature space relative to the sample population size, Team Whole-Sale Modelers applied principal component analysis to restrict the feature space to several dozen principal components that accounted for approximately 40% of the variation in the over 100,000 phenotype measurements. Third, the phenotype measurements associated with each sample point were stochastic. To increase the robustness of DE to stochastic variation, Team Whole-Sale Modelers used the reduced phenotype measurements to iteratively train a random forest estimator for each parameter [40]. The random forest parameter estimate was initialized to the best performing individual in the DE population. Individuals generated by the random forest parameter estimation procedure were then used to seed the DE population.

**Fig 5:**
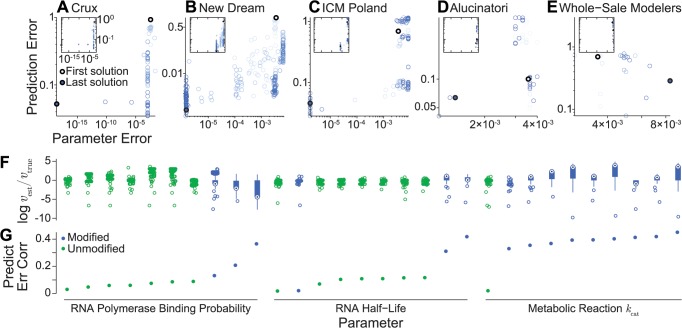
Individual team parameter estimation performance. Parameter and prediction error trajectories of the top five scoring teams (**A**: Crux, **B**: New Dream, **C**: ICM Poland, **D**: Alucinatori, **E**: Whole-Sale Modelers). For comparison, insets depict each team’s trajectory using common x- and y-scales defined in A. Submissions are colored by time. Light blue indicates each team’s first submission; dark blue indicates each team’s last submission. Light and dark blue dots indicate the first and last submissions, respectively. Trajectories show that Team Crux employed a deterministic algorithm which steadily improved their performance over the competition, whereas the other teams employed stochastic algorithms which randomly sampled the parameter space, resulting in non-monotonically increasing performance. **F**: Box plots of the estimated values of each unknown parameter. Blue indicates the 15 modified parameters; green indicates the 15 unknown and unmodified parameters. **G**: Correlations between the estimated value of each unknown parameter and the prediction error.

Fig 5E illustrates Team Whole-Sale Modelers’ performance. Their approach improved only the prediction error and not the parameter error.

In addition, a few teams used reduced physical models to estimate specific model parameters from specific in silico data. Team CU estimated the RNA polymerase promoter binding probabilities from the RNA polymerase ChIP-seq data using the DNA-seq data to correct for DNA copy number differences along the chromosome from the oriC to terC. Team CU then used the estimated binding probabilities to estimate the unknown RNA half-lives from the RNA expression data. Team Alucinatori estimated the unknown reaction turnover rates using short time scale simulations of the metabolic submodel. Team Alucinatori refined the parameters by matching metabolic fluxes between the reduced and full models.

Four teams focused on the artificial problem of estimating the unknown parameters using parameter error information from BitMill. Although these four teams did not focus on the real-world parameter estimation problem, their methods may be applicable to the real-world parameter estimation problem. Further work is needed to assess their methods on real-world parameter estimation.

We analyzed the teams’ error trajectories to better understand their relative merits, including their performance and efficiency. We found that Team Crux’s derivative-based approach not only achieved the lowest parameter error but also was the most efficient strategy, arriving at the top solution using the smallest number of model iterations among the top performing teams (Fig 5A). In contrast to Team Crux, Teams New Dream, ICM Poland, and Alucinatori used methods that wandered through the error landscape, causing them to slowly and inefficiently approach the true parameter values (Fig 5B–5D).

Next, we inspected the submitted parameter values to gain further insight into how participants explored the parameter space (Fig 5F). We found that RNA polymerase binding probabilities and metabolic reaction turnover rates had the largest range of submitted values, suggesting that the teams focused on exploring these parameters. We also calculated each parameter’s correlation with the prediction error (Fig 5G) to better understand why participants focused on exploring these parameters. We found that the metabolic turnover rate parameters were the most correlated with the prediction error. However, further analysis is needed to understand whether the prediction error was simply correlated with the turnover rate parameters because participants changed these parameters the most significantly, or because the prediction error is most highly sensitive to the turnover rate parameters.

## Discussion

We organized the DREAM8 parameter estimation challenge to develop new parameter estimation techniques for whole-cell models. To mimic the real-life problem of estimating whole-cell model parameters, we constructed a mutant in silico strain by modifying the parameters of a whole-cell model of *M*. *genitalium* and asked participants to identify the modified parameter values given the model’s structure and several simulated experimental data sets. We provided participants with the BitMill cloud computing service to simulate the model free of charge and encouraged participants to form teams.

The challenge represented a simplified version of the parameter estimation problem faced in real-world whole-cell modeling. Participants were asked to identify a subset (2%) of the model’s parameters, a common problem researchers face when developing a model of a part of a larger system. In addition, participants were given consistent in silico experimental data representing experiments obtained using a single strain with a single experimental condition. In contrast, real whole-cell models must be identified using heterogeneous data originating from multiple organisms, laboratories, and experimental conditions. Participants were also given much more training data than is typically available experimentally. In real-world applications, it is infeasible to comprehensively characterize each perturbation. Typically only a limited amount of data is available for each perturbation. For example, only growth rates are available for each *M*. *genitalium* single gene disruption strain. Lastly, the in silico experimental data contained no measurement noise, only the intrinsic stochastic variation present in the model.

We established the challenge as a competition rather than as a conventional research project for two reasons. First, we wanted to expand the whole-cell modeling community by providing researchers an opportunity to contribute to the field. Second, many groups have shown that competitions can quickly and inexpensively produce high-quality scientific results [32–34,41–43]. The challenge successfully attracted researchers to the emerging field of whole-cell modeling, including researchers from a broad range of scientific disciplines. We hope these new researchers will help advance whole-cell modeling.

Ten teams participated in the challenge. Anecdotally, participants reported that free availability of the BitMill cloud computing service was critical to the challenge’s success. Several teams stated that they would not have had sufficient time or resources to set up computing clusters to compete the challenge, and that they would not have participated without the free and user-friendly BitMill service. Overall, BitMill enabled more scientists to participate and enabled those scientists to focus more of their time on the scientific content of the challenge rather than on duplicating efforts to establish computational infrastructures. We therefore believe that shared cloud computing platforms such as BitMill could improve participation and performance in other DREAM challenges and other crowdsourced scientific projects.

The participants primarily pursued two families of approaches. Four teams tried to solve the artificial problem of identifying the unknown model parameters using the parameter error metric and derivative-based approaches. These derivative-based approaches can also be effective for real-world parameter estimation of small, deterministic models where gradient calculations are tractable and where good estimates of the true parameter values are available such that the optimization procedure is seeded in the attractor basin of the global optimum. For these reasons, derivative-based approaches alone are not well suited to estimating stochastic, computationally expensive models. For whole-cell models, derivative approaches must be used in combination with other techniques such as surrogate modeling or model reduction.

Five other teams tried to solve the real-world problem of identifying the unknown parameters using only the experimental data and the prediction error metric. These teams used a variety of parameter estimation techniques to reduce the prediction error metric, led by Team Whole-Sale Modelers, who developed a novel combination of DE and random forests. Notably, Team Whole-Sale Modelers identified the directions in which the parameters were modified with 80% (12 of 15 modified parameters) accuracy.

In addition, a few teams pursued strategies based on reduced physical models. These teams tried to estimate the RNA polymerase promoter binding probabilities from the RNA polymerase ChIP-seq data, use this information to estimate the RNA half-lives from the RNA microarray data, and use the protein expression data, metabolic fluxes, and FBA metabolic submodel to estimate the reaction turnover rates.

We decided to provide participants parameter distance information to give participants qualitative feedback on how far their models were to the true parameter values. We did not intend for participants to use this information to solve the challenge. We incorrectly believed that teams would not use this information because this information is not available in real-world biological parameter estimation applications. Unfortunately, we did not learn that participants were using this information to solve the artificial parameter error optimization problem until the last week of the challenge, at which point we felt it was too late to change the structure of the challenge. In hindsight, we should have anticipated that participants would use the parameter error information because the challenge is organized as a competition with the artificial end goal of “winning” rather than the real-world end goal of creating knowledge.

Despite the artificial nature of this challenge, it generated valuable new ideas about how to best identify whole-cell models. One team developed a novel combination of DE and random forests, and two teams explored model reduction strategies. Interestingly, none of the teams pursued distributed optimization or automatic differentiation, which have been used in other fields for computationally expensive models.

The challenge also generated useful information about parameter identifiability. The challenge highlighted the degeneracy of the parameter error, meaning that multiple parameter sets can produce similar errors due to degeneracies in phenotypic subspaces, and that comprehensive data is required to make the parameters practically identifiable [44–47]. This degeneracy in phenotypic subspaces is consistent with observations of many other biophysical systems [48–55]. Modelers must avoid creating structurally unidentifiable parameters that can never be estimated.

## Lessons Learned

In addition, we learned several valuable lessons about how to best organize challenges. Most importantly, we learned that participants will use all available information. Organizers should never provide information that could be used to side step the challenge.

We also learned that it is important to assess the feasibility of the challenge beforehand. This should be achieved by assessing the feasibility theoretically, as well as by asking a small number of colleagues to beta test the challenge before public release. For parameter estimation challenges, this means rigorously assessing the practical identifiability of the unknown parameters using the training data that will be provided to the participants and limiting the challenge to structurally identifiable parameters.

Third, we learned that participants will only share their approaches if they believe they can win a prize. This means that organizers should only release performance statistics prior to prize selection if participants have similar performance; otherwise, only participants who perceive they have a chance to win a prize will share their methods, and the community will never be able to learn from other methods that were explored but never shared. Furthermore, to encourage all participants to share their approaches, regardless of their numerical success, organizers should randomly award prizes simply for participating.

Lastly, we learned that to maximize participation, organizers must make every effort to minimize the prior knowledge and resources required to participate in the challenge. For computational challenges, one way to minimize the required resources is to provide free, preconfigured computational resources. We believe this is especially important for computationally expensive challenges that require complicated and expensive computing clusters. Furthermore, we found that modeling challenges must provide participants a clear, thorough, and accessible description of the mathematical model and its parameters.

## Conclusion

Overall, the challenge confirmed that whole-cell model parameter estimation is a formidable problem. Significant work remains to develop efficient parameter estimation methods suitable for high-dimensional, nonlinear whole-cell models. Nevertheless, the challenge successfully expanded the whole-cell modeling community and initiated an important dialogue about how to best estimate whole-cell model parameters.

Going forward, several parameter estimation innovations are needed to enable researchers to achieve fully accurate models of complex organisms. First, researchers need to develop automated methods for constructing reduced models which are tractable to numerical optimization. Researchers should pursue both statistical and physics-based reduced models. Ideally, these models will take advantage of the unique temporal and population average structure of most experimental cell biology data.

Second, researchers must develop simulation engines that quickly execute whole-cell models. This will enable researchers to more accurately identify parameters by enabling them to quickly explore parameter combinations. This can be accomplished by developing a simulation engine that executes multiple submodels simultaneously and that parallelizes the execution of each individual submodel.

Third, researchers must develop distributed optimization algorithms that quickly explore the parameter space. Individual workers should communicate so that workers each learn from each other. These parallel optimization methods will enable researchers to find ensembles of highly optimal solutions.

Fourth, researchers must develop visualizations that highlight differences among model simulations. This will help researchers design experiments to select among otherwise equivalently scoring parameters sets. In turn, this will help researchers discover and characterize new biological mechanisms.

Lastly, researchers need to develop new high-throughput experimental technologies that characterize single-cell variation and temporal dynamics. High-throughput measurements have enabled whole-cell modeling by greatly increasing their practical identifiability. However, currently researchers still have to estimate variance parameters from systems data. New technologies could enable researchers to more easily estimate variance parameters, as well as test variance predictions.

We are optimistic that collaborative efforts such as DREAM will produce these new tools. These tools will enable researchers to build more accurate models of more complex organisms, starting with more complex bacteria such as *E*. *coli* followed by single-celled eukaryotes such as *Saccharomyces cerevisiae*, multicellular eukaryotes such as *Caenorhabditis elegans*, and lastly humans. In turn, these new models will open new avenues for rationally designing microorganisms with unprecedented capabilities and ultimately enable physicians to design personalized medical therapies.

### DREAM8 Parameter Estimation Challenge Consortium

#### Team 9

Yucheng Hu^1^

**1** Zhou Peiyuan Center of Applied Mathematics, Tsinghua University, Beijing, China

#### Team Alucinatori

Michael Baron^1^, Kevin Bryson^1^

**1** University College London, London, United Kingdom

#### Team Crux

Andreas Raue^1,2^, Bernhard Steiert^1,2^, Jens Timmer^1–4^, Clemens Kreutz^1,2^

**1** Institute for Physics, University of Freiburg, Freiburg, Germany

**2** Freiburg Center for Systems Biology (ZBSA), University of Freiburg, Freiburg, Germany

**3** Freiburg Institute for Advanced Studies (FRIAS), University of Freiburg, Freiburg, Germany

**4** BIOSS Centre for Biological Signalling Studies, University of Freiburg, Freiburg, Germany

#### Team CU

Brandon Barker^1,3^, Elijah Bogart^2^, Yiping Wang^1,3^, Dhruva Chandramohan^1^, Lei Huang^1^, Kelson Zawack^1,3^, Alexander A. Shestov^4^

**1** Department of Biological Statistics and Computational Biology, Cornell University, Ithaca, New York, United States of America,

**2** Department of Physics, Cornell University, Ithaca, New York, United States of America,

**3** Tri-institutional Training Program in Computational Biology & Medicine, Weill Cornell Graduate School of Biomedical Sciences, Cornell University, New York, New York, United States of America,

**4** Division of Nutritional Sciences, Cornell University, Ithaca, New York, United States of America

#### Team DBI-Guesstimators

Hiren Makadia^1^, Danielle DeCicco^2^

**1** Daniel Baugh Institute, Thomas Jefferson University, Philadelphia, Pennsylvania, United States of America,

**2** Graduate Program in Cell & Developmental Biology, Thomas Jefferson University, Philadelphia, Pennsylvania, United States of America

#### Team Hurricane

Alex Yin^1^, Mengqing Wang^1^, Shuai Cheng Li^1^

**1** Department of Computer Science, City College of Hong Kong, Hong Kong, China

#### Team ICM Poland

Marcin Świstak^1,2^, Mateusz Cygan^1,5^, Denis Kazakiewicz^3,4^, Miron B. Kursa^1^, Przemyslaw Korytkowski^6^, Dariusz Plewczynski^1,4†^

**1** Interdisciplinary Centre for Mathematical and Computational Modelling, University of Warsaw, Warsaw, Poland,

**2** Faculty of Mathematics, Informatics and Mechanics, University of Warsaw, Warsaw, Poland,

**3** Wrocław University of Technology, Wrocław, Poland,

**4** The Centre for Innovative Research, Medical University of Białystok, Białystok, Poland

**5** Faculty of Automatic Control, Electronics and Computer Science, Silesian University of Technology, Gliwice, Poland,

**6** Faculty of Computer Science and Information Technology, West Pomeranian University of Technology, Szczecin, Poland

^†^Present address: The Jackson Laboratory for Genomic Medicine, University of Connecticut Health Center, Farmington, Connecticut, United States of America and Yale University, New Haven, Connecticut, United States of America

#### Team New Dream

Jichen Yang^1^, Yajuan Li^2,3^, Hao Tang^1^, Tao Wang^1^, Yueming Liu^4^, Yang Xie^1^, Guanghua Xiao^1^

**1** Quantitative Biomedical Research Center, Department of Clinical Sciences, University of Texas Southwestern Medical Center at Dallas, Dallas, Texas, United States of America,

**2** Laboratory of Disease Genomics and Individualized Medicine, Beijing Institute of Genomics, Chinese Academy of Sciences, Beijing, China,

**3** Department of Immunology and Internal Medicine, University of Texas Southwestern Medical Center, Dallas, Texas, United States of America,

**4** Department of Mathematics, University of Texas at Arlington, Arlington, Texas, United States of America

#### Team Uniandes

Julian Bello^1^, David Octavio Botero Rozo^2^, Silvia Johana Cañas-Duarte^3^, Juan Camilo Castro^3^, Fabio Gomez^1^, Ivan Valdes^1^, Laura González Vivas^4^, Adriana Bernal^2^, Juan Manual Pedraza Leal^4^, Silvia Restrepo^2^, Alejandro Reyes Muñoz^3^

**1** Universidad de los Andes, Bogotá, Colombia,

**2** Mycology and Plant Disease Laboratory, Universidad de los Andes, Bogotá, Colombia,

**3** Department of Biological Sciences, Universidad de los Andes, Bogotá, Colombia,

**4** Department of Physics, Universidad de los Andes, Bogotá, Colombia

#### Team Whole-Sale Modelers

Alex H. Williams^1,2^, Jeremy D. Zucker^3^

**1** Department of Biology, Brandeis University, Waltham, Massachusetts, United States of America,

**2** Volen Center for Complex Systems, Brandeis University, Waltham, Massachusetts, United States of America,

**3** Broad Institute, Cambridge, Massachusetts, United States of America

## Supporting Information

S1 Table Whole-cell model quantitative parameters. (PDF)

S2 Table Mutant RNA polymerase promoter binding probabilities. (PDF)

S3 Table Mutant RNA half-lives. (PDF)

S4 Table Mutant reaction turnover numbers. (PDF)
