# Supplementary material for: Summary of the DREAM8 Parameter Estimation Challenge: Toward Parameter Identification for Whole-Cell Models
Source: PLoS Comput Biol. 2015 May 28;11(5):e1004096. doi: 10.1371/journal.pcbi.1004096 (PMC4447414; doi:10.1371/journal.pcbi.1004096)
Supplement: S1 Table — (PDF) [file pcbi.1004096.s001.pdf]

Table S1. Whole-Cell Model Quantitative Parameters.

Type	Count
**External stimuli value**	10
**Metabolite extracellular concentration**	83
**Metabolite intracellular concentration**	73
**Reaction turnover number**	434
**RNA half-life**	343
**RNA polymerase promoter binding probability**	335
**Transcription factor fold-change effect**	30
**Other**	154
