# Supplementary material for: Summary of the DREAM8 Parameter Estimation Challenge: Toward Parameter Identification for Whole-Cell Models
Source: PLoS Comput Biol. 2015 May 28;11(5):e1004096. doi: 10.1371/journal.pcbi.1004096 (PMC4447414; doi:10.1371/journal.pcbi.1004096)
Supplement: S2 Table — (PDF) [file pcbi.1004096.s002.pdf]

Table S2. Unknown Mutant RNA Polymerase Promoter Binding Probabilities. Empty entries indicate unmodified parameters.

		RNA Polymerase-Promoter Binding Probability	
**Transcription Unit**	**Genes**	**Wild-Type**	**Mutant**
**TU003**	MG005-009	0.0046	
**TU012**	MG025	0.0037	0.0022
**TU028**	MG048	0.0011	
**TU070**	MG113	0.0006	
**TU184**	MG277	0.0085	0.0050
**TU209**	MG306	0.0010	
**TU245**	MG344	0.0015	
**TU272**	MG376	0.0022	
**TU306**	MG428	0.0010	0.0002
**TU319**	MG448	0.0009	
