# Supplementary material for: Summary of the DREAM8 Parameter Estimation Challenge: Toward Parameter Identification for Whole-Cell Models
Source: PLoS Comput Biol. 2015 May 28;11(5):e1004096. doi: 10.1371/journal.pcbi.1004096 (PMC4447414; doi:10.1371/journal.pcbi.1004096)
Supplement: S3 Table — (PDF) [file pcbi.1004096.s003.pdf]

Table S3. Unknown Mutant RNA Half-lives. Empty entries indicate unmodified parameters.

Transcription Unit	Genes	Half-life (s)	
		Wild-Type	Mutant
**TU003**	MG005-009	209	203
**TU011**	MG022-024	245	
**TU027**	MG046-047	170	97
**TU069**	MG111-112	187	89
**TU180**	MG270-272	401	
**TU203**	MG299	174	
**TU233**	MG330-333	253	
**TU260**	MG357	216	
**TU294**	MG407	282	
**TU307**	MG429-431	219	
