# Supplementary material for: Summary of the DREAM8 Parameter Estimation Challenge: Toward Parameter Identification for Whole-Cell Models
Source: PLoS Comput Biol. 2015 May 28;11(5):e1004096. doi: 10.1371/journal.pcbi.1004096 (PMC4447414; doi:10.1371/journal.pcbi.1004096)
Supplement: S4 Table — (PDF) [file pcbi.1004096.s004.pdf]

**Table S4. Unknown Mutant Reaction Turnover Numbers.** Empty entries indicate unmodified parameters.

| | | $k_{cat}$ ($s^{-1}$) | |
|---|---|---|---|
| **Name** | **Reaction** | **Wild-Type** | **Mutant** |
| **Acetate kinase** | AcP + ADP ↔ Ac + ATP | 100.59 | 53.15 |
| **Cytidylate kinase** | ATP + CMP ↔ ADP + CDP | 103.25 | 8.00 |
| **Enolase** | G2P ↔ $H_2O$ + Pep | 300.87 | 573.46 |
| **Fructose-bisphosphate aldolase** | Fdp ↔ DHAP + GAP | 23.34 | 26.06 |
| **Glucose-6-phosphate isomerase** | G6P ↔ F6P | 1218.79 | 245.80 |
| **Methionine adenosyltransferase** | ATP + $H_2O$ + Met → AdoMet + Pi + PPi | 0.11 | 0.06 |
| **Phosphotransacetylase** | Ac-CoA + Pi ↔ AcP + CoA | 1620.91 | 146.41 |
| **Pyruvate dehydrogenase** | CoA+NAD+Pyr ↔ Ac-CoA+$CO_2$+NADH | 1128.31 | 456.00 |
| **Thiamine kinase** | ATP + dTMP ↔ ADP + dDTDP | 0.07 | |
| **Triose-phosphate isomerase** | DHAP ↔ GAP | 816.67 | 53.65 |
